# Key Salivary Proteins Implicated in the Inhibition of *Staphylococcus Aureus* Biofilms by Chlorogenic Acid

**DOI:** 10.1055/s-0046-1816083

**Published:** 2026-02-16

**Authors:** Shuichiro Kamei, Toshiki Uematsu, Kaori Ochiai, Masanori Saito, Hidenobu Senpuku

**Affiliations:** 1 Department of Microbiology and Immunology, Nihon University Dental School at Matsudo, Chiba, Japan

**Keywords:** α-amylase, *S. aureus*, chlorogenic acid, salivary components, biofilm

## Abstract

**Objective:**

*Staphylococcus aureus* ( *S. aureus* ), a causative agent of infective endocarditis, is found in the oral cavity. Chlorogenic acid (CA) is a polyphenol compound and a secondary metabolite produced by tea, coffee, etc., which are selected as inhibitors of biofilm formation. This study was conducted to clarify how coated human saliva controls the inhibition of *S. aureus* biofilms by CA.

**Materials and Methods:**

Bacteria were cultivated with various concentrations of CA on nonsalivary components or salivary components-coated 96-well microtiter plates in tryptic soy broth supplemented with 0.25% sucrose and various concentrations of NaCl.

**Results:**

The biofilm formation of *S. aureus* was induced on noncoated plates but inhibited by more than 2.5 mg/mL CA on salivary components-coated plates in 0.125 M or 0.25 M NaCl. The degree of *S. aureus* biofilm inhibition was affected by coating with different salivary components. In addition, the salivary components, such as α-amylase, upregulated the biofilm formation and were associated with the inhibitory effects of CA on biofilm formation.

**Conclusion:**

Exposure to CA through the consumption of coffee and tea may be an inhibitor of biofilm formation by *S. aureus* via interaction with salivary α-amylase in individuals who consume food and drinks containing high concentrations of salt.

## Introduction

Oral microorganisms communicate with the environment when forming oral biofilms that cause tooth decay and periodontal disease on the surface of teeth. They metabolize nutrients and form biofilms by adhering, proliferating, and aggregating polysaccharides on the surface of teeth covered with saliva and gingival crevicular fluid. 1 2 When biofilms form, the microorganisms within them become resistant to antimicrobial substances and gain the ability to survive in the oral cavity for long periods of time. 3 4 In developed countries, the proportion of elderly people is increasing due to changes in the balance between birth rates and aging, and the number of bedridden elderly people is also increasing. 5 The mortality rate from aspiration pneumonia among elderly individuals is increasing and is associated with oral biofilms. Older adults are more likely to experience systemic diseases related to oral microorganisms. 6 7 Systemic diseases such as diabetes and hypertension also contribute to increased rates of infectious diseases. 8

*Staphylococcus aureus* ( *S. aureus* ) is an opportunistic pathogen that is widely found on human skin, in the nasal mucosa, and in the oral cavity. 9 *S. aureus* , which has also been detected in the oral cavity, has been reported as a causative agent for systemic diseases, which can have mild to life-threatening implications. 10 This bacterium is associated with systemic infections such as food poisoning, infectious endocarditis, osteomyelitis, and pneumonia. 10 11 *S. aureus* , a bacterium resistant to high salt concentrations, induces both single-species and mixed-species biofilms with *S. mutans* under high salt conditions. 12

Biofilm formation by *S. aureus* is promoted in culture media containing specific concentrations of NaCl. 13 14 This phenomenon is considered to depend on the salt concentration, but environmental conditions such as the presence of saliva composition may also control biofilm formation by *S. aureus* . The detailed mechanism remains unclear. This control may be associated with the survival ability of *S. aureus* under harsh conditions containing high salt concentrations.

Chlorogenic acid (CA) is a polyphenolic compound and a secondary metabolite produced by the consumption of tea, coffee, and traditional Chinese medicine. 15 16 CA directly targets bacterial cell walls and cell membranes, causing irreversible osmotic damage, and thus has potential as an effective antibacterial agent. 17 18 19 Recently, MSCRAM has been defined as proteins associated with the cell wall surface of *S. aureus* , and it has been suggested that it may bind to components in saliva. 20 Therefore, MSCRAM may serve as targets for the effects of CA on the biofilm formation of *S. aureus* under the interaction between MSCRAM and salivary components. CA effectively inhibits biofilm formation while minimizing cytotoxicity 21 and is useful as a safe inhibitor for pathogen infection. Various factors affecting the saliva composition of the oral cavity may contribute to and influence the formation of *S. mutans* and other microorganisms' biofilms. 22 23 24 The purpose of this study was to identify which components in saliva contribute to the formation of single-species biofilms of *S. aureus* and to determine the role of these factors under salinity conditions, for the biofilm inhibition by CA. Studying how CA acts as an inhibitor for the biofilm formation of *S. aureus* under the interaction between bacteria and saliva components is important for the care of elderly patients with systemic diseases.

## Materials and Methods

### Bacterial Strains and Culture Conditions

*S. aureus* Cowan I (Methicillin-susceptible *S. aureus* ; MSSA) was cultured and maintained at 37°C in a 5% CO _2_ atmosphere (Gas Pack: Mitsubishi Gas Chemical Company, Tokyo, Japan) in brain–heart infusion (BHI) broth (Becton Dickinson and Company, Franklin Lakes, New Jersey, United States) for experimental purposes. The growth of *S. aureus* was evaluated by measuring the turbidity in BHI containing CA as the absorbance at 595 nm (OD _595_ ).

### Collection of Human Saliva

Saliva samples were collected from three healthy subjects aged 25 to 53 years (K, Y, and M) over a period of 5 minutes while chewing paraffin gum. They are healthy subjects with no underlying medical conditions who have not taken antibiotics for at least 3 months. The amount of *S. aureus* infection in their oral cavity was low, at 200 CFU/mL or less. The samples were centrifuged at 10,000 × g for 10 minutes at 4°C, filtered and sterilized using 0.22 µm and 0.45 µm Millex-GP filter units (Merck kGaA), and then added to a 96-well polystyrene microtiter plate (Sumitomo Bakelite, Tokyo, Japan) for use in a biofilm formation assay. Bacteria, including *S. aureus* , were completely removed by filter sterilization because the bacteria never grew in the growth medium, including collected saliva. The use of human saliva for plate coating in this study was approved by the Ethics Committee of Nihon University School of Dentistry in Matsudo (IRB approval number: EC21-029). Prior to subject registration, written informed consent was obtained from each subject in accordance with the World Medical Association's Declaration of Helsinki.

### Subtraction Assay of Human Salivary Components using *S. aureus* Whole Cells for α-Amylase

*S. aureus* was cultured overnight in 30 ml of BHI. The cells were centrifuged at 6,000 × g, washed twice with sterile PBS, and then subjected to a subtraction assay with various concentrations of human saliva. One ml of human saliva (sample M) of α-amylase was suspended with *S. aureus* whole cells inactivated with 4% paraformaldehyde and incubated for 1 hour at 37°C. After incubation, whole cells were removed by centrifugation at 6,000 × g, and salivary components attached to whole cells were removed from the salivary samples. The subtracted saliva sample was used for SDS—PAGE, and silver staining.

### Biofilm Formation Assay

Biofilms of *S. aureus* were cultured overnight at 4°C in 96-well polystyrene microtiter plates (Sumitomo Bakelite Co., Ltd., Tokyo, Japan), precoated with sterile human saliva. After washing with sterile PBS, a biofilm formation assay was performed according to a modified method. 25 *S. aureus* Cowan I was inoculated at a ratio of 1:100 into 200 µl of trypsin soy broth (TSB) containing 0.25% sucrose in the presence of 0.125 M NaCl. This concentration of NaCl was found to be an enhancer of the biofilm formation of *S. aureus* . 12 The plates were then incubated at 37°C under aerobic conditions with 5% CO _2_ for 16 h. After cultivation, planktonic cells were removed, and the biofilm was washed with distilled water (DW), and adherent cells were stained with 0.25% safranin for 15 minutes to measure the extent of biofilm formation. 25 After washing twice with DW, safranin was eluted from the biofilm with 70% (vol/vol) ethanol. The degree of biofilm formation was measured using the color intensity of the extracted safranin solution. Biofilm formation was quantified by measuring the absorbance of the stained biofilm at 492 nm.

### Observing Live and Dead Cells During Biofilm Formation

The biofilms were stained with the FilmTracer Live/Dead Biofilm Viability Kit (Molecular Probes, Inc., Eugene, Oregon, United States), and SYTO 9 and propidium iodide were added to the biofilms, each at a final concentration of 5 µM. A live/dead staining assay was performed in TSB with 0.25% sucrose, where red indicates dead cells, green indicates live cells, and yellow indicates a mix of live and dead cells.

The biofilms were incubated with the dyes at room temperature for 20 to 40 minutes before being imaged via confocal laser scanning microscopy (CLSM; LSM700 Meta NLO, Carl Zeiss Inc., Jena, Germany). Two-dimensional (2D) images were acquired with a Plan-Apochromat 10 ×/0.45 M 27 objective lens. The confocal images of the biofilms were evaluated using ZEN (Carl Zeiss, ZEN2008) analysis software.

### SDS—PAGE

Prior to electrophoresis analysis, human saliva and subtraction saliva were diluted to equal volumes or SDS—PAGE buffer (0.06 M Tris-HCl [Amersham Pharmacia Biotech, Buckinghamshire, United Kingdom], pH 6.8; 20% glycerol [Wako Pure Chemical Industries, Ltd., Osaka, Japan]; 0 or 1% [wt/vol] SDS [Wako]; 0 or 1% 2-mercaptoethanol [2-ME, Merck kGaA]; and 0.0012% bromophenol blue [Wako]). SDS—PAGE samples were heated at 100°C for 5 minutes immediately before loading onto the gel. The SDS—PAGE samples were then applied to a 12.5% polyacrylamide gel (e-PAGEL, ATTO Corp., Tokyo, Japan) containing 0.025 M Tris-HCl, 192 mM glycine (Wako), and 0% or 0.1% (wt/vol) SDS. Protein electrophoresis separation was performed at 40 mA for 80 minutes. The gel was stained with Coomassie Brilliant Blue (CBB, Wako) and silver stain (2D-SILVER STAIN II, Cosmo Bio Co., Ltd., Tokyo, Japan).

### Measurement of α-Amylase

Human salivary samples and the salivary M sample with *S. aureus* whole cells subtracted were quantitatively measured by an α-amylase measurement kit (Kikkoman Inc., Tokyo, Japan).

### Statistical Analysis

The extent of biofilm formation is expressed as the means ± standard deviations (SDs). In the biofilm assay, differences among various concentrations of CA were determined via one-way analysis of variance (ANOVA) and the Bonferroni correction (IBM SPSS Statistics 24, IBM Corporation, Armonk, New York, United States). A *p* -value less than 0.05 was considered to indicate statistical significance. All experiments were repeated independently three times.

## Results

Effects of CA on the growth of *S. aureus* were observed in the presence and absence of 0.125 M NaCl. Five mg/mL CA significantly inhibited the growth of *S. aureus* in the presence of 0.125 M NaCl but not in other concentrations of NaCl ( Fig. 1A ). CA has collaborated with NaCl for the growth inhibition. To clear the biofilm formation in the presence of 0.125 M NaCl and CA, the biofilm formation was first observed in a noncoated plate. The biofilm formation was significantly increased at 0.125 M NaCl and 2.5 mg/mL CA but not significant as compared with other concentrations of NaCl ( Fig. 1B ). To clear the contribution of NaCl to biofilm formation compared with no NaCl, various concentrations of NaCl were added in presence or absence of 2.5 mg/mL CA, which was showed a highest biofilm formation level, and the biofilm formations were observed. The biofilm formation was increased with and without CA by the addition of NaCl ( Fig. 1C ). Finally, CA showed growth-inhibitory activity only at 0.125 M NaCl, suggesting that NaCl enhances the sensitivity of *S. aureus* to CA. However, it was found that CA did not affect biofilm formation of *S. aureus* induced by NaCl on a noncoated plate.

**Fig. 1:**
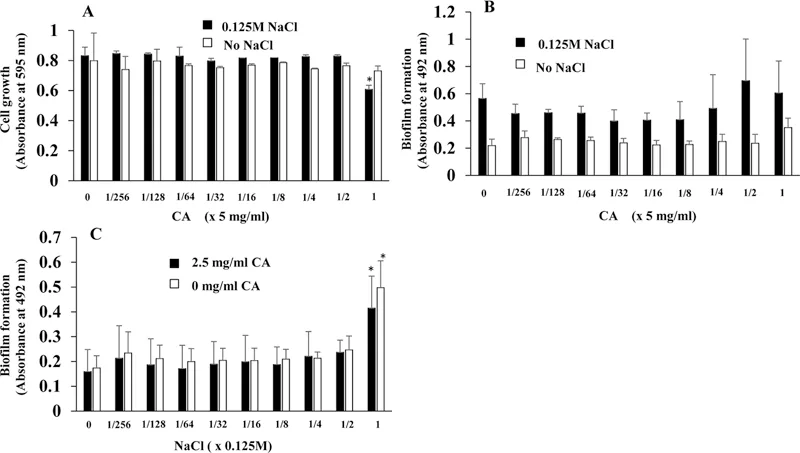
Effects of CA and NaCl on the growth and biofilm formation of *S. aureus* with noncoated plates. Growth ( **A** ) and biofilm formation ( **B, C** ) of *S. aureus* Cowan I were quantitively measured at 0, 1/2 ^8^ , 1/2 ^7^ , 1/2 ^6^ , 1/2 ^5^ , 1/2 ^4^ , 1/2 ^3^ , 1/2 ^2^ , 1/2 ^1^ , and 1/2 ^0^ × 5 mg/mL CA dilutions in BHI and TSB with 0.25% sucrose, respectively, or 0, 1/2 ^8^ , 1/2 ^7^ , 1/2 ^6^ , 1/2 ^5^ , 1/2 ^4^ , 1/2 ^3^ , 1/2 ^2^ , 1/2 ^1^ , and 1/2 ^0^ × 0.125 M NaCl in TSB with 0.25% sucrose ( **C** ). The data are presented as the means ± SDs of three independent experiments. The data are presented as the means ± SDs of three independent experiments. An asterisk indicates that there is a significant difference between the two groups (* *p* < 0.05, vs. control: no CA or no NaCl).

To clarify the effects of coating of human saliva on the biofilm formation of *S. aureus* , the biofilm formation of *S. aureus* Cowan I was measured on the plate coated with human saliva (K, Y, and M). The biofilm formation of *S. aureus* was strongly induced on the M saliva-coated plate compared with that of the other saliva samples (Y and K) and was significantly greater in sample M than in samples K and Y at 1/100 and 1/200 dilutions of the cell suspension cultured overnight in BHI ( Fig. 2A ) in the salinity condition. The biofilm formation of *S. aureus* was significantly inhibited in a dose-dependent manner by CA on sample M-coated culture plate but not on sample Y-coated plates ( Fig. 2B ). The biofilm formation was significantly inhibited at 5 mg/mL CA on sample K-coated plate. The coating of human saliva samples changed the effect of CA on biofilm formation in noncoating conditions.

**Fig. 2:**
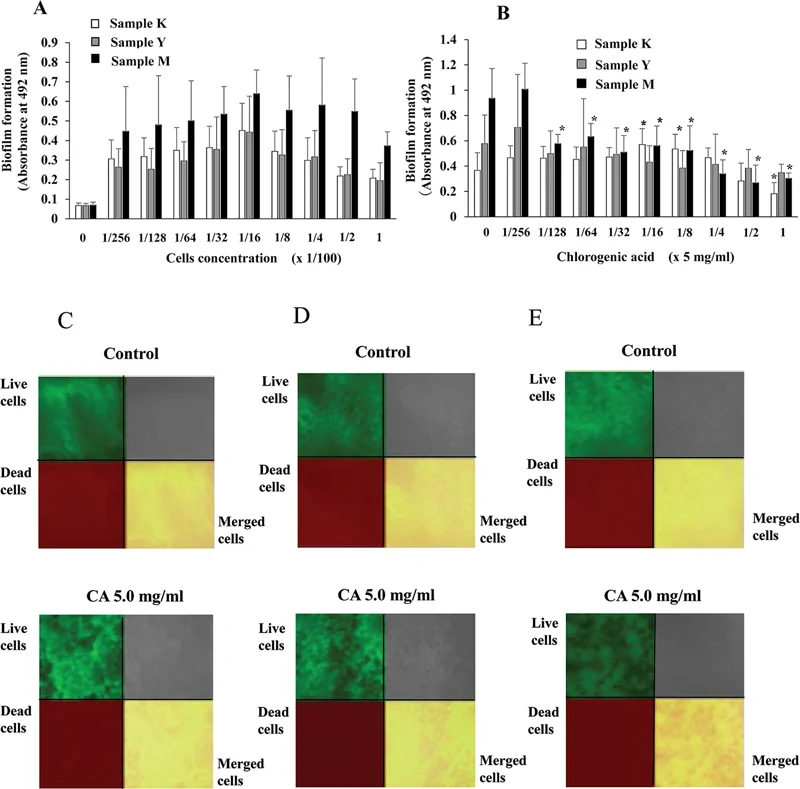
Effects of human salivary coating on the biofilm formation of *S. aureus* with human saliva K, Y, and M-coated plates. Biofilm formation of *S. aureus* Cowan I were quantitively measured at 0, 1/2 ^8^ , 1/2 ^7^ , 1/2 ^6^ , 1/2 ^5^ , 1/2 ^4^ , 1/2 ^3^ , 1/2 ^2^ , 1/2 ^1^ , and 1/2 ^0^ bacterial cells dilutions (1/100) grown in BHI ( **A** ) or 0, 1/2 ^8^ , 1/2 ^7^ , 1/2 ^6^ , 1/2 ^5^ , 1/2 ^4^ , 1/2 ^3^ , 1/2 ^2^ , 1/2 ^1^ , and 1/2 ^0^ × 5 mg/ml CA ( **B** ), in TSB with 0.25% sucrose. The data are presented as the means ± SDs of three independent experiments. An asterisk indicates that there is a significant difference between the two groups (* *p* < 0.05, CA concentrations vs. no CA). Effects of CA on the biofilm formation of *S. aureus* were observed by a confocal microscope. *S. aureus* was inoculated in TSB with 0.25% sucrose with various concentrations of 5.0 mg/mL CA in the biofilm formation assay with human saliva K ( **C** ), Y ( **D** ), and M ( **E** ) coated plates. These biofilm formations were treated via live/dead staining and observed via confocal microscopy. Representative data from more than three independent experiments were presented in the pictures.

To elucidate the mechanisms of biofilm inhibition by CA, live/dead staining was performed, and the biofilms were observed by confocal microscopy. Compared with the control treatment, treatment with 5.0 mg/mL CA resulted in the formation of *S. aureus* biofilms that had gaps on the surface, which gathered live bacteria and formed clumps on the K-, Y-, and M-coated plates ( Fig. 2C–E ). CA had bactericidal effects but did not completely kill bacterial cells during growth as evidenced by the slightly inhibition of growth with CA ( Fig. 1A ). The coating of the saliva M sample has the ability to spread biofilms by live cells, increase the volume of the biofilm in the condition without CA ( Fig. 2E ) and, however, is significantly inhibited by CA ( Fig. 2E ).

To research the interaction of the components in saliva samples with *S. aureus* , a subtraction assay of the components in saliva samples using *S. aureus* whole cells fixed with 4% paraformaldehyde was performed. The attached components with *S. aureus* whole cells were removed by centrifugation, and the removed components were confirmed by SDS—PAGE and silver staining. The intensity of the protein band at approximately 62 kDa, which is estimated as the molecular weight of α-amylase, was reduced after subtraction by S. aureus whole cells ( Fig. 3A ). α-Amylase is the main protein in salivary components. By magnifying the photograph, a decrease in the intensity of the protein band at approximately 62 kDa was highlighted after the subtraction assay using *S. aureus* whole cells ( Fig. 3B ). α-Amylase has the ability to interact with components of extra polysaccharide (EPS) within biofilms formed by *S. aureus* . 26 To compare α-amylase among saliva samples quantitatively, the activities were measured by an α-amylase measurement kit. The activities of α-amylase were significantly greater in sample M than in samples K and Y ( Fig. 4A ). To confirm the effects of the subtraction of *S. aureus* whole cells on the decrease in the α-amylase band in sample M, the activities of α-amylase were measured in samples before and after subtraction in saliva sample M. The activity of α-amylase was significantly lower in samples after subtraction in saliva sample M than in samples before subtraction ( Fig. 4B ). Therefore, α-amylase may be a key component for biofilm formation of *S. aureus* and biofilm inhibition by CA under high-salinity conditions.

**Fig. 3:**
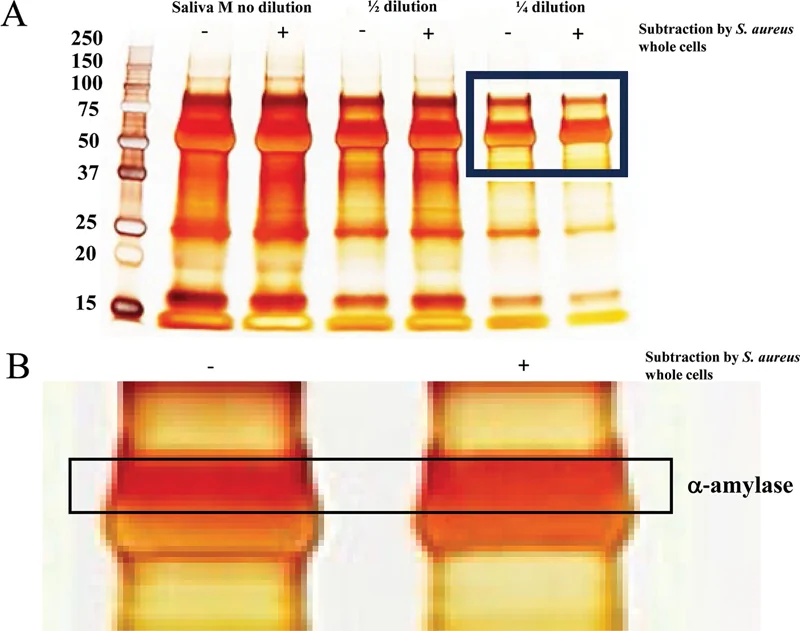
Effects of subtraction by *S. aureus* whole cells on the proteins in saliva samples. The nonsubtracted saliva samples and the *S. aureus* whole cells-subtracted saliva sample were applied for SDS-PAGE, and silver staining was performed to observe the expression of proteins. The intensity of the protein band around 62 kDa ( **A** ) was highlighted by magnifying the photograph ( **B** ), and a decrease in the intensity of the protein band around 62 kDa was found after the subtraction assay using *S. aureus* whole cells. Representative data from more than three independent experiments were presented in the pictures.

**Fig. 4:**
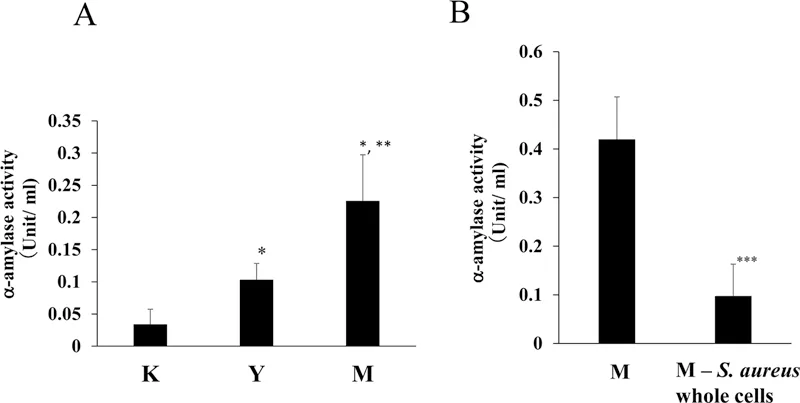
Effects of subtraction of *S. aureus* whole cells on the activities of α-amylase in saliva samples. Activities of α-amylase in the nonsubtracted saliva samples (K, Y, and M), ( **A** ) the nonsubtracted sample *M* and *S. aureus* whole cells-subtracted saliva M sample ( **B** ) were observed by the measurement kit. The data are presented as the means ± SDs of three independent experiments. An asterisk indicates that there is a significant difference between the two groups (* *p* < 0.05, sample M vs. K, ** *p* < 0.05, sample M vs. Y, *** *p* < 0.05, nonsubtraction vs. subtraction).

To clarify the interaction between *S. aureus* and α-amylase, commercial α-amylase from B. subtilis was coated on a culture plate for the biofilm formation assay. The biofilm formation levels of *S. aureus* were significantly greater on the α-amylase-coated plate than on the noncoated plate ( Fig. 5 ). To clarify the effects of different concentrations of NaCl, which induce the biofilm formation of *S. aureus* , 13 0.25 M NaCl was used for the biofilm formation assay. Biofilm formation was temporarily increased by the addition of 2.5 mg/mL CA to the noncoated and α-amylase-coated plates ( Fig. 5 ). However, biofilm formation was significantly inhibited by 5 mg/mL CA on noncoated and α-amylase-coated plates. Therefore, α-amylase may be one of the inducers of salivary components in *S. aureus* biofilms under salinity conditions, and the biofilm formation was also inhibited by CA.

**Fig. 5:**
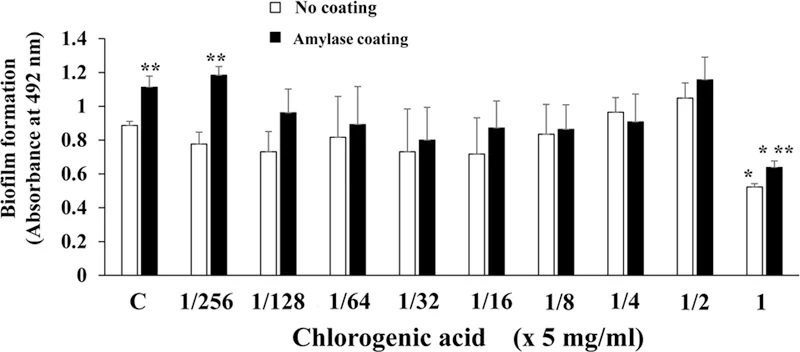
Effects of coating of a-amylase on the growth and biofilm formation of *S. aureus* . Biofilm formation of *S. aureus* Cowan I was quantitatively measured at 0, 1/2 ^8^ , 1/2 ^7^ , 1/2 ^6^ , 1/2 ^5^ , 1/2 ^4^ , 1/2 ^3^ , 1/2 ^2^ , 1/2 ^1^ , and 1/2 ^0^ × 5 mg/mL CA on the culture plate coated and noncoated with a-amylase in TSB with 0.25% sucrose, involved in 0.250 NaCl. The data are presented as the means ± SDs of three independent experiments. An asterisk indicates that there is a significant difference between the two groups (* *p* < 0.05, CA vs. no CA, ** *p* < 0.05, coating of a-amylase vs. noncoating).

## Discussion

CA is among the polyphenols that are attracting attention due to their usefulness for maintaining health, and are among the components that give coffee beans their sour taste, and are found in large concentrations in green coffee beans. 27 It is also found in vegetables such as potatoes, eggplants, and carrots, and in fruits such as cherries, peaches, and apples, and is a substance that humans have been consuming since ancient times. CA has attracted attention as an antioxidant and has been shown to be effective in preventing stroke, breast cancer, diabetes, Parkinson's disease, and obesity. As phenolic compounds with a hydroxyl group in the ortho position, they have also been shown to have anti-infective and anti-inflammatory effects.

In this study, CA has slightly collaborated with the 0.125 M NaCl condition for the growth inhibition of *S. aureus* on a noncoated plate. Furthermore, CA suppressed biofilm formation by *S. aureus* in environments in which all saliva samples (K, Y, M) were coated in the same concentration environments, suggesting that CA may have the potential to prevent many systemic diseases in humans, such as infectious endocarditis and aspiration pneumonia. The intake of food and drinking involving CA may be particularly effective in suppressing *S. aureus* infection in people who prefer a high-salt diet. Five mg/mL CA is a greater concentration than normally found in coffee, so to see results, individuals would need to drink as strong a coffee as possible. Five mg/mL CA suppressed the growth of *S. aureus* ( Figs. 1 and 2 ) and killed one of the biofilm cells, but other concentrations were more effective at suppressing biofilm formation than growth under the interaction between *S. aureus* and salivary M component ( Figs. 2 and 3 ). Including CA concentration management, this is considered an efficient biofilm suppression method that effectively controls coffee intake. However, the efficacy of these CAs applies to the MSSA used in this study and may differ for MRSA (Methicillin-resistant *S. aureus* ). MRSA infection continues to sustain as a major public health threat worldwide, especially in the elderly. 28 Further investigation is required to clarify the protective effects of CA on the oral infection and the biofilm formation of *S. aureus* MRSA.

The amount of *S. aureus* biofilm formed was affected by the composition of the coating saliva. In particular, the saliva M sample contained a large amount of α-amylase, and it is likely that α-amylase increased the amount of *S. aureus* biofilm formed. Compared with other saliva samples, α-amylase is an enzyme and cell-surface-associated protein from human saliva and bacteria that catalyzes the conversion of complex sugars to monosaccharides, which are required for bacterial colonization and survival of pathogens on environmental surfaces. 29 It randomly cleaves the 1,4-a-D-glycosidic linkages between adjacent glucose units. However, the human salivary α-amylase sequence is different from that of α-amylase proteins from other sources. 30 It has the ability to interact with components of EPS within biofilms formed by *S. aureus* , 31 inhibits cell-to-cell communication and cell-to-cell surface adhesion, and breaks preexisting *S. aureus* aggregates and biofilms. 26 31 In contrast, it was suggested that the a-amylase coating induced the biofilm formation of *S. aureus* under high-salinity conditions. α-Amylase is an essential structural component of the in situ pellicle layer on tooth surfaces in humans, 32 33 34 but its functional role is uncertain. The inhibition of biofilm formation may be lost by physically blocking the active site while attaching to the plate surface because coating α-amylase from B. subtilis, which has greater inhibitory activity than salivary α-amylase, induced more biofilm formation of *S. aureus* on the culture plate ( Fig. 5 ). It serves as a specific receptor for the adherence of numerous oral bacteria. 35 36 37 α-amylase is a key structural component of pellicles to which *S. aureus* has adapted. However, potential residual bacterial components or DNA in saliva may also influence interactions between *S. aureus* and the tooth surface. As it was practically difficult to clarify these in this study, it remains an issue for future research.

Elevated levels of α-amylase are associated with stress responses and could be indicative of the body's reaction to ongoing pathological processes, such as inflammation and malignant transformation. 38 39 The level of salivary α-amylase increases during stress, supporting bacterial colonization on the tooth surface. Low levels of salivary α-amylase activity have been detected, probably because a low-starch diet results in low caries prevalence. 40 Salivary α-amylase is a notable protein of saliva that plays a role in the host response by acting as an inhibitory factor against microorganisms. Sánchez et al showed that the sympathetic system is activated by the inflammatory process in periodontitis and upregulates the production of salivary α-amylase to increase the salivary defense potential. 41 The production is systemically controlled during stress responses and may be associated with both the colonization of *S. aureus* and the defense against *S. aureus* infection. Previous studies have shown that α-amylase works to inhibit biofilm formation by *S. aureus* , but when the biofilm is physically attached, it is considered to act to promote adhesion. When attached, the inhibitor's function may be inhibited, and only the target recognition function may work. However, the induction of the biofilm on the α-amylase-coated surface might be inhibited by CA in the salinity condition. However, since this study was conducted with only three saliva donors, it is necessary to increase the number of subjects or examine saliva components other than α-amylase in future studies to enhance accuracy.

These findings suggest that the consumption of coffee beverages as leisure drinks may suppress infections caused by opportunistic bacteria such as *S. aureus* . The concentration of CA in coffee beverages is relatively low at 2 mg/mL at most, and it is necessary to take into account changes in CA, which are easily affected by changes in concentration due to the drip method and heat caused by roasting, as the position of the hydroxyl group is easily affected. It is necessary to recognize the factors affecting CA concentration and intake levels, and ultimately conduct clinical research. Improving the method of ingestion may provide safer and more effective preventive measures against oral and systemic infections in individuals who consume food and drinks containing high concentrations of salt.

## Conclusion

Exposure to CA through consumption of coffee and tea may be an inhibitor of biofilm formation by *S. aureus* via interaction with salivary α-amylase in individuals who consume food and drinks containing high concentrations of salt.
